# HIV-1 Nef Is Transferred from Expressing T Cells to Hepatocytic Cells through Conduits and Enhances HCV Replication

**DOI:** 10.1371/journal.pone.0099545

**Published:** 2014-06-09

**Authors:** In-Woo Park, Yan Fan, Xiaoyu Luo, Myoung-Gwi Ryou, Jinfeng Liu, Linden Green, Johnny J. He

**Affiliations:** 1 Department of Cell Biology and Immunology, University of North Texas Health Science Center, Fort Worth, Texas, United States of America; 2 Pharmacology and Neuroscience, University of North Texas Health Science Center, Fort Worth, Texas, United States of America; 3 Microbiology and Immunology, Indiana University School of Medicine, Indianapolis, Indiana, United States of America; University of Rome Tor Vergata, Italy

## Abstract

HIV-1 infection enhances HCV replication and as a consequence accelerates HCV-mediated hepatocellular carcinoma (HCC). However, the precise molecular mechanism by which this takes place is currently unknown. Our data showed that infectious HIV-1 failed to replicate in human hepatocytic cell lines. No discernible virus replication was observed, even when the cell lines transfected with HIV-1 proviral DNA were co-cultured with Jurkat T cells, indicating that the problem of liver deterioration in the co-infected patient is not due to the replication of HIV-1 in the hepatocytes of the HCV infected host. Instead, HIV-1 Nef protein was transferred from nef-expressing T cells to hepatocytic cells through conduits, wherein up to 16% (average 10%) of the cells harbored the transferred Nef, when the hepatocytic cells were co-cultured with nef-expressing Jurkat cells for 24 h. Further, Nef altered the size and numbers of lipid droplets (LD), and consistently up-regulated HCV replication by 1.5∼2.5 fold in the target subgenomic replicon cells, which is remarkable in relation to the initially indolent viral replication. Nef also dramatically augmented reactive oxygen species (ROS) production and enhanced ethanol-mediated up-regulation of HCV replication so as to accelerate HCC. Taken together, these data indicate that HIV-1 Nef is a critical element in accelerating progression of liver pathogenesis via enhancing HCV replication and coordinating modulation of key intra- and extra-cellular molecules for liver decay.

## Introduction

Due to the shared routes of infection, HIV-1/HCV co-infection is common, with 15∼30% of all HIV-1-infected persons estimated to be co-infected with HCV [1], [2], [3]. In the co-infected patients, HIV-1 is known to accelerate every stage of HCV-mediated liver disease progression, such as two-fold acceleration of fibrosis and five-fold higher risk of cirrhosis-related liver complications, etc. [4], [5], and thus infection in Western countries has become a leading cause of morbidity and mortality in HIV-1-infected individuals [6], [7], [8]. However, the molecular details regarding how co-infection of HIV-1 and HCV brings about a more severe deterioration of the liver than a single infection of HCV are unknown at present.

One established feature with respect to liver disease is that co-infection of HIV-1 and HCV generates higher loads of HCV than do HCV mono-infected controls [9], [10], [11]. However, hepatocytes do not support productive replication of HIV-1 [12], [13], regardless of several reports claiming that HIV-1infects liver cells [14], [15], [16], [17], [18], [19], suggesting that up-regulation of HIV-1-mediated HCV replication could be attributed by intra- and extra-cellular direct or indirect interactions of HCV-infected hepatocytes with specific HIV-1 viral proteins, such as Tat and envelope (Env) protein. It is very well known that HIV-1 Tat protein is diffusible [20], and therefore this protein secreted from the HIV-1 infected cells could be diffused into hepatocytes to dysregulate replication of HCV and expression of hepato-cellular genes to expedite liver disease. Tat itself is also known to enhance hepatocarcinogenesis in transgenic mice [21], [22]. It is also possible that Env glycoprotein (gp120) shed from the infected CD4+ cells or embedded within HIV-1 virus particles could interact with CXCR4 or CCR5 co-receptor molecules expressed on the surface of hepatocytes [23], [24] and trigger signaling cascades to modulate expression of viral genes of HCV and/or cellular genes of hepatocytes. This is supported by the findings that the interaction of gp120 with CXCR4 on the surface of hepatocytes enhanced HCV replication in the replicon system, and the effect was abrogated with neutralizing antibodies against CXCR4 [25]. Interaction of Env with CXCR4 also induces apoptosis of hepatocytes together with HCV E2, and modulates signaling cascades of inflammatory cytokines involved in hepatic inflammation [26], [27], [28], [29]. However, these data need to be further confirmed, since a recent report by Iser at al [17] indicates that CXCR4, CCR5 and CD4 are not expressed in hepatic cells.

Recent studies indicate that HIV-1 Nef protein plays a pivotal role in the formation of various HIV-1-associated diseases through its transfer from HIV-1-infected cells to HIV-1-uninfected bystander T lymphocytes [30], [31] and even to HIV-1-nonsusceptible B cells [31] via intercellular conduits. Many of the known functions of Nef are relevant to the process of intercellular transmission through conduits. Since Nef is myristoylated [32], it targets the cell membrane and is involved in cytoskeletal rearrangement, organelle formation and immunological synapse destabilization [33], [34]. Nef also inhibits ruffle formation, but induces the synthesis of long, thin filopodium-like protrusions [30], events which are important for protein trafficking. Thus, it is reasonable to assume that HIV-1 Nef expressed from HIV-1 infected T cells, macrophage/monocytes, and/or dendritic cells travels to hepatocytes through conduits and alters the course of HCV-mediated liver disease. However, it is completely unknown whether HIV-1 Nef is transferred from the HIV-1-infected cells to hepatocytes in the infected host, and if so, what the pathobiological impacts of transfer of Nef on hepatocytes are.

This study demonstrates that HIV-1 Nef expressed in T lymphocytes can be transferred to hepatocytic cell lines and up-regulate HCV replication by modulating intracellular lipid distribution. Further, Nef enhanced ethanol-mediated up-regulation of HCV replication and augmented ROS production, providing critical molecular clues with respect to how co-infection of HIV-1 and HCV exacerbates HCV-mediated hepatocellular disease.

## Materials and Methods

### Cells, Plasmids, and Reagents

Human hepatocytic cell line, Huh7.5.1 cells and subgenomic HCV replicon cells known as RLuc cells, expressing Renilla luciferase reporter (RLuc) as well as nonstructural genes (from NS3 to NS5B) flanked by the 5′- and 3′-untranslated region (UTR) of HCV [35], were cultured in complete DMEM in the presence or absence of 200 µg/ml G418, respectively, unless otherwise specified. Construction and maintenance of Jurkat T cells expressing GFP or HIV-1 Nef.GFP have been described previously [36]. Methyl-β-cyclodextrin (MβCD) was purchased from Sigma, Inc. pEGFP-N3 was purchased from Clontech biotechnology (Clontech Inc. CA), and pEGFP-Nef.GFP was constructed by placing the PCR amplified nef gene in between the EcoRI and BamHI restriction sites of the plasmid pEGFP-N3, so that the Nef.GFP fusion protein is expressed. Inducible GFP- and Nef.GFP-expressing Jurkat cell lines using a Tet-off system (Clontech) were described in a previous report [37], and the clones were maintained in complete RPMI1640 plus 200 µg/ml Hygromycin (Boehringer Mannheim), 200 µg/ml G418 (Invitrogen, NY), and 2 µg/ml tetracycline (Sigma Chemical Co. MO). Plasmid constructs, pGL2B-FAS-1500 [38], LDLTLuc [39], and pSynSRE-luc [40], were kindly provided by Dr. Timothy Osborne, Sanford-Burnham Medical Research Institute, Orlando, FL. MitoSox Red for superoxide and H_2_DCFDA (DCFDA) for ROS production assays were purchased from Invitrogen Inc., (Eugene, OR) and Molecular Probes, Inc., (Eugene, OR), respectively. All research involved in the manuscript has been approved by the authors' Institutional Review Board of the University of North Texas Health Science Center, and written informed consent has been obtained for such research.

### Production and infection of pseudotyped HIV luciferase reporter viruses

HIV-1 luciferase reporter viruses pseudotyped with envelopes of HIV-1 strains of 89.6, HXBc2, and vesicular stomatitis virus (VSV) were generated, as described previously [41], [42]. Infection was performed by incubating the target cells with pseudotyped viruses equivalent to 2000 cpm reverse transcriptase (RT) activity at 37°C for 2 h. The cells were then removed of excessive virus, incubated in complete culture medium for a further 48 h, and harvested for the luciferase activity assay. To investigate whether HIV-1 replicates in hepatocytes, the HXBc2 strain of HIV-1 corresponding to 10,000 cpm was used to infect 1×10^6^ Huh7.5.1 cells. Culture supernatants were collected every other day to monitor the amount of virus within the supernatants, and reverse transcriptase (RT) activity in the culture supernatants was determined, as described [42].

### Co-cultivation

To investigate Nef transfer from nef-expressing cells to target hapatocytes via conduits, RLuc subgenomic replicon cells cultured in the 6 well plate or on the cover glass for flow cytometry or confocal microscopy, respectively, were stained with 1 mM SP-DilC18, according to the manufacturer's directions (Sigma), and the stained cells were co-cultured with GFP- or nef.GFP-expressing Jurkat cells for 24 h. Jurkat cells were then removed by gentle washing with PBS, and the hepatocytic cells on the cover glass were employed for the confocal microscopy, while the cells in the 6 well plate were trypsinized, washed with PBS, and used for the flow cytometry to quantify green and red positive cells.

To study Nef transfer through exosomes, RLuc cells stained with SP-DilC18 were cultured with the culture supernatants or exosomes isolated from the culture supernatants of GFP- or nef.GFP-expressing Jurkat cells, as described [43], for 24 h, and transfer of Nef to RLuc was analyzed, as described above.

### Transfection and reporter gene assay

The indicated cells grown on a cover slip or in a 24-well plate were transfected with the indicated amount of each plasmid using the lipofectamine 2000 transfection reagent (Invitrogen). Total amount of plasmid DNA was adjusted with pCDNA3 or pEGFP-N3 to eliminate potential discrepancies resulting from unequal amounts of transfected DNA, and pCMV-βGal was co-transfected to standardize transfection efficiency. The Luciferase assay was performed using *Renilla* or *Firefly* Luciferase assay kits (Promega, WI), following the manufacturer's instructions. To elucidate the effect of lipids on HCV replication, HCV RLuc cells cultured on 24 well plates were treated with the serum-free (SF) DMEM for 24 hours, followed by 1 hour treatment with the indicated concentration of MβCD in the SF-DMEM. To examine the combinatorial effect of Nef and MβCD on HCV replication, RLuc cells were transfected with the Nef-expressing plasmid (PEGFP-Nef.GFP), as described above, and 48 hour post-transfection, the transfected cells were treated with the indicated concentration of MβCD. All presented data were standardized based on the amount of lysate and transfection efficiency, and all luciferase assays were repeated at least three times in duplicate or triplicate format.

### ROS assays

Reactive oxygen species were detected by two different methods. MitoSox Red was used to detect primarily superoxide generated in mitochondria. Briefly, cells in 96 well plate were transfected with 0.1 µg pCDNA3-nef.Myc, using the lipofectamine 2000 transfection reagent. Two days after transfection, cells were washed with PBS and incubated with MitoSox Red (5 µmol/L) for 10 min at 37°C. The fluorescent signal was visualized and photographed on a Zeiss fluorescence microscope. ROS was assessed by a fluorometric assay using DCFDA. Again, cells were transfected with the indicated amount of pCDNA3-nef.Myc, as described above, and 48 h after transfection, cells were loaded with10 µmol/L DCFDA for 30 min at 37°C. After washing with PBS, regular culture media was added, and fluorescence was measured at 495 nm excitation and 530 nm emission wavelengths.

### Ethanol treatment

RLuc cells were cultured onto 3.5 cm plates for 24 h, and 4 µg of nef.GFP-expressing plasmid, pCMV-nef.GFP, was transfected into the RLuc culture using the lipofectamin transfection method. Twenty-four hours after transfection, cells were treated with the indicated concentration of ethanol, placed in a larger dish containing an identical concentration of ethanol as the media, sealed the larger dish with parafilm, and cultured for 48 hrs. Cells were then harvested, and luciferase activity in the harvested cells was measured, as described above.

### Oil Red O staining and Immunofluorescence analyses

Cells grown on the polylysine-coated cover slips were transfected with the indicated plasmids, as described above. For Oil Red O (ORO) staining, cells were washed twice with PBS, fixed in 4% paraformaldehyde for 5 min, and subjected to ORO staining, as described [44], [45], and microscopic images were taken using an immunofluorescence microscope. For confocal microscopy, the subgenomic replicon RLuc cells were red-labeled with 1 µM SP-DilC18(3) (1,1′-dioctadecyl-6,6′-di(4-sulfophenyl)-3,3,3′,3′-tetramethylindocarbocyanine), according to the manufacturer's protocol (Invitrogen) and co-cultured with the indicated number of gfp- or nef.GFP-expressing Jurkat cells for the indicated time. Transfer of GFP from Jurkat cells to the target RLuc cells was then analyzed by confocal microscopy. For the microscopic analysis of Nef transfer from HIV-1 replicating Jurkat cells to RLuc cells, the indicated number of wild type or nef-deletion mutant HIV-1-infected Jurkat cells was co-cultured with the SP-DilC18(3)-labeled RLuc cells grown on the cover slip for 24 h, and Nef protein that transferred from Jurakat to RLuc cells was visualized using mouse anti-Nef antibody (Santa Cruz) followed by anti-mouse FITC.

### Flow cytometry analyses

Jurkat cell clones were induced to express GFP or Nef.GFP proteins, as described above. Subgenomic replicon RLuc cells were labeled with 1 µM SP-DilC18(3), and the stained cells were then washed twice with PBS, resuspended in PBS, and analyzed by flow cytometry.

### Immunohistochemistry

Immunohistochemical (IHC) staining was performed according to the protocol provided by Abcam. Briefly, a paraffin-embedded specimen from HCV-monoinfected and HIV-1/HCV-co-infected patients were deparaffinized and rehydrated by a series of xylene/ethanol washes, and antigen was retrieved using a heat-induced epitope retrieval method in the antigen retrieval buffer (Dako, CA). The specimens were then permeabilized with 0.2% TritonX-100 and stained with anti-Nef antibody (Santa Cruz, CA) followed by horse-reddish peroxidase (HRP)-conjugated secondary antibody and tyramide labeling.

### Data analysis

All values were expressed as mean ± SEM of triplicate samples or experiments, unless otherwise noted. Comparisons among groups were made using two-tailed Student's t-test. A p value of <0.05 was considered statistically significant (*), and p<0.01 highly significant (**).

## Results

### HIV-1 did not infect human hepatocytic cells

**Figure 1:**
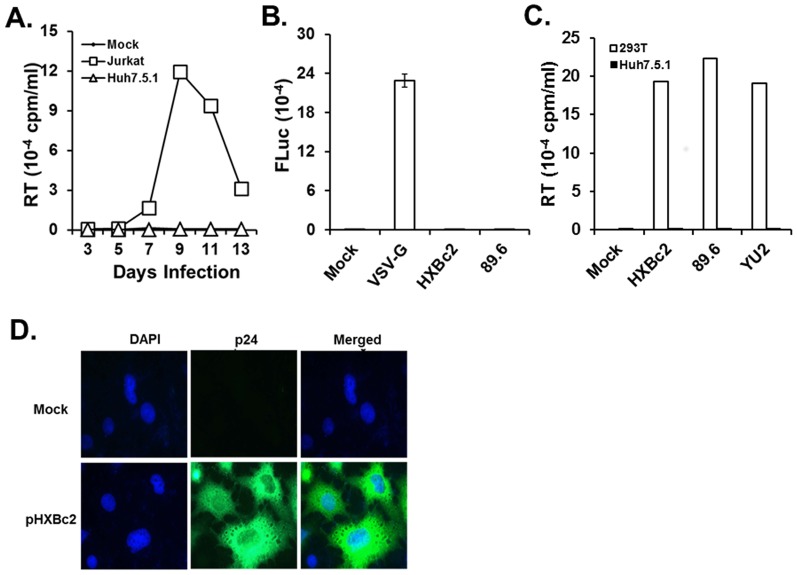
Replication of HIV-1 in human hepatocytes. (A) Replication of HIV-1 in Huh7.5.1. Culture supernatants of RLuc and Jurkat T cells were collected for RT assay, and RT activity in the supernatants was measured at the indicated time points. Jurkat T cells were included as a positive control. (B) Entry of pseudotype HIV-1 into Huh7.5.1. HIV-1 pseudotyped with Env of HXBc2 or 89.6 strain and VSV with G protein were used to infect target Huh7.5.1 cells, and a Luc assay was performed with the cell lysates prepared from the infected cells, as described in the text. (C) Replication of HIV-1 from transfected cells. Proviral DNA of the indicated strain of HIV-1 was transfected into Huh7.5.1 cells (closed bar) or 293T cells (open bar), and accumulation of infectious progeny viruses from the transfected cells was monitored by measuring RT activity in the culture supernatants. (D) Expression of p24. Huh7.5.1 cells were transfected with proviral DNA of the HXBc2 strain of HIV-1, and expression of p24 in the transfected cells was examined by fluorescence microscopy. Green and blue colors indicate p24 protein and nucleus, respectively.

To investigate the infectivity of HIV-1 in human hepatocytes, cell-free HIV-1 was added to Huh7.5.1 on a 6 well plate, and virus replication was monitored at the indicated time point by measuring RT activity in the clarified culture supernatants, as described [42]. As a positive control, the same amount of HIV-1 was infected into susceptible Jurkat T cells. The data showed that HIV-1 was replicated productively in Jurkat T cells, but not in Huh7.5.1 cells, indicating that human hepatocytic cells do not support HIV-1 replication (Fig. 1A). Similarly, HepG2 cells did not support productive replication of HIV-1 (data not shown). The lack of HIV-1 replication in human hepatic cell lines could be due to the inability of the virus to enter into hepatocytes. To investigate this possibility, HIV-1 viruses pseudotyped with Env of HXBc2 or 89.6 strain of HIV-1 or with VSV were infected into Huh7.5.1 cells, and luciferase (Luc) activity in the infected cells was assayed, as described [41]. The results showed that high Luc activity was detected from Huh7.5.1 cells infected with VSV-, but not from HIV-1-Env pseudotyped viruses (Fig. 1B), indicating that the lack of replication of HIV-1 in Huh7.5.1 cells was due to restriction of HIV-1 entry into human hepatocytes. Since transfection of provirus into cells bypasses the entry step, we examined whether infectious progeny viruses were generated from the hepatocytic cells transfected with HIV-1 proviral DNA. Our results showed that replication of HIV-1 in 293T cells was robust, while no RT activity was detected in the culture supernatants from the transfected hepatocytic cells (Fig. 1C), although viral protein, p24 was detected in Huh7.5.1 cells by fluorescence microscopy from the transfected provirus (Fig. 1D). Western blot analysis showed that even the ratio of the amount of p24 to that of the precursor Gag in Huh7.5.1 transfected with HXBc2, YU2, and 89.6 strains of HIV-1 proviral DNA was significantly lower than that in 293T cells (data not shown), indicating that limited processing of the precursor Gag to p24 could also contribute to the restricted replication of HIV-1 in Huh7.5.1 cells. In addition to this, co-cultivation of Jurkat cells with the transfected Huh7.5.1 cells failed to show any sign of virus replication for an extended period of time (data not shown), confirming that Huh7.5.1 cells were not susceptible to HIV-1 replication. Similar results were obtained using the subgenomic replicon, RLuc (data not shown). Taken together, these data lead to the conclusion that hepatocytes are not susceptible to HIV-1 replication.

### Nef protein was transferred to RLuc cells through conduits

**Figure 2:**
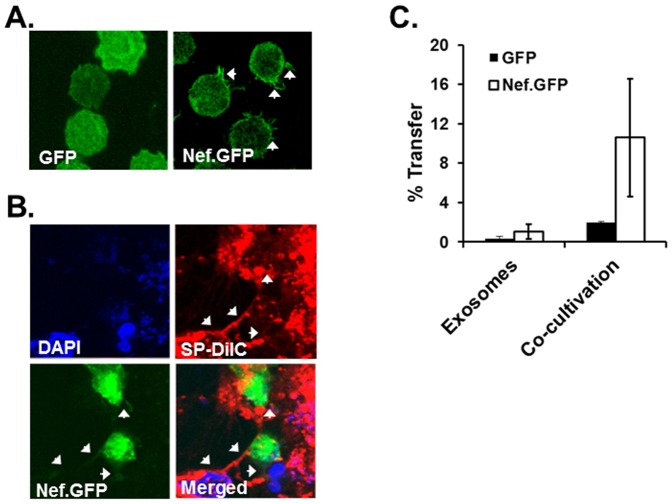
Transfer of Nef protein from Jurkat T cells into hepatocytes. (A) Formation of conduits in nef.GFP-expressing Jurkat T cells. gfp- or nef.GFP-expressing Jurkat T cells were adhered to the poly-lysine coated cover slip, and cell morphology was investigated by confocal microscopy. Arrows indicate conduits. (B) Extension of conduits from nef.GFP-expressing Jurkat cells to hepatocytes. RLuc replicon cells grown onto a poly-lysine coated cover slip were stained with SP-DilC18(3), and the stained cells (red) were co-cultured with nef.GFP-expressing Jurkat cells (green) for 24 hr in complete DMEM media. Extension of conduits from Jurkat cells to RLuc cells (arrows) was then analyzed by confocal microscopy. (C) Quantification of Nef transferred from Jurkat to RLuc. (Co-cultivation) RLuc (1×10^6^ cells) stained with SP-DilC18(3) was co-cultivated with the same number of gfp- (closed bar) or nef.GFP (open bar)-expressing Jurkat cells for 24 hr in a 6 well plate, as described above. After Jurkat cells were removed by gentle wash with PBS, RLuc cells were detached from the plate by trypsinization, washed twice, and subjected to flow cytomery to quantify the transfer of GFP to the target RLuc cells. (Exosomes) The same number of RLuc cells was cultured with exosomes in the culture supernatants of gfp- or nef.GFP-expressing Jurkat cells, and the transfer of Nef in the exosomes to the hepatocytes was quantified in a similar manner, as described. Bar graph indicates % presentation of green positive cells to total red positive cells (RLuc cells).

Since HIV-1 does not productively replicate, but is nonetheless known to exacerbate HCV-mediated hepato-disease, we hypothesized that HIV-1 Nef is transferred to hepatocytes by conduit formation or exosome secretion to communicate with target cells. Thus, we first investigated whether Nef is transferred from nef-expressing cells to target hepatocytic cells using Jurkat cell line expressing Tetracycline (Tet)-inducible nef. To this end, we first studied whether nef-expression enables the cells to protrude conduits or not. Our data showed that conduits were not extended from gfp-expressing Jurkat cells used as controls in this study, while significant extension of conduits were detected from nef.GFP-expressing Jurkat cells (arrows in Fig. 2A), consistent with the previous reports [30], [31]. Next, we investigated whether Nef.GFP was transferred from Jurkat cells to target RLuc cells through conduits. Confocal images showed that nef.GFP-, but not GFP-, expressing Jurkat cells extended conduits to the target replicon cells (arrows in Fig. 2B). Furthermore, the conduits displayed the green color of Nef.GFP in the red color, used as a marker of RLuc, indicating that Nef.GFP protein was able to transfer from Jurkat T cells to RLuc (Fig. 2B). Transfer of Nef protein to RLuc cells was further confirmed by flow cytometry. Again, after co-cultivation of gfp- or nef.gfp-expressing Jurkat cells with SP-DilC18(3)-labeled RLuc cells for 24 hr, RLuc cells fluorescing positive for both green and red colors were quantified by flow cytometry. Our data indicated that 5 to 16% (average 10%) of RLuc cells were consistently positive for both Red and Green color, while approximately 2% of hepatocytes were both positive, when RLuc was co-cultured with control GFP-expressing Jurkat cells (Fig. 2C). It is reported that HIV-1 Nef can also be transferred from HIV-1-infected cells to the non-susceptible cells through exosomes [46]. Thus, possibility of Nef transfer through exosomes was investigated by concentration of exosomes and virus particles followed by 6-18% Optiprep density gradient fractionation from HIV-1-infected Jurkat cells. Acetylcholine esterase activity assay together with Western blot analyses with anti-p24 and anti-Nef antibodies of each fraction indicated that fractions harboring HIV-1 or Nef protein were completely separated from those with exosomes, demonstrating that both HIV-1 virus particles and nef protein are not secreted as with exosomal complexes (manuscript in preparation). Further, our flow cytometric analysis showed that there is no concrete evidence of transfer of Nef from Jurkat cells to hepatocytic cells through exosomes, when RLuc or Huh7.5.1 were cultured with the exosomes in the culture supernatants of nef.GFP Jurkat clone (Fig. 2C). Taken together, these data indicate that HIV-1 nef was transferred to target hepatocytes through conduits.

### Nef expressed in HIV-1-infected T cells was transferred to the target cells

**Figure 3:**
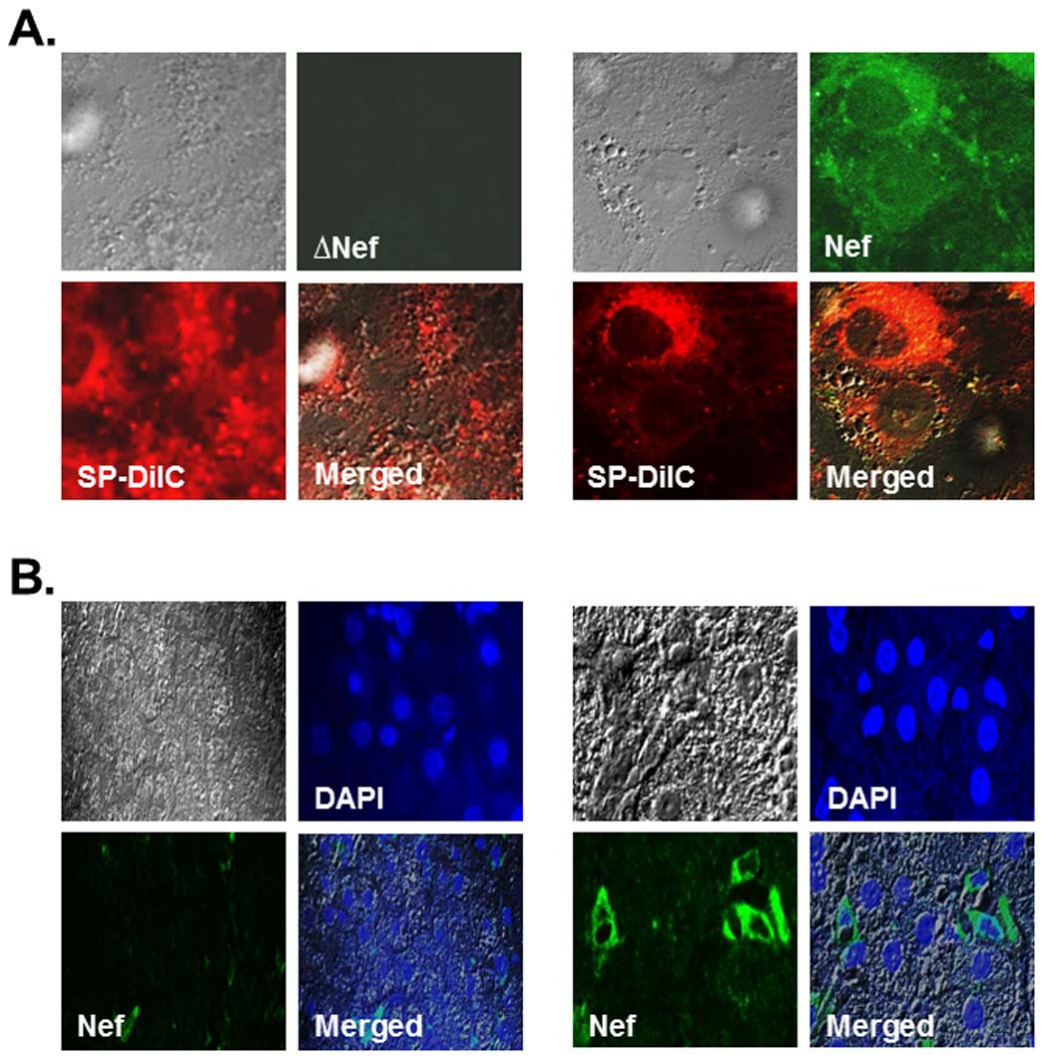
Confocal analyses. (A) Transfer of Nef. Transfer of Nef protein from wild type- (right panel) or Δnef-HIV-1 infected (left panel) Jurkat cells to the target, RLuc, was investigated by confocal microscopy. The transferred Nef protein (green) from the infected Jurkat cells to RLuc was visualized using anti-nef antibody followed by anti-mouse Alexa488. Red indicated Nef protein and RLuc cells stained with SD-DilC18. (B) Immunohistochemistry of primary hepatocytes. Left and right panels showed liver specimen from HCV- and HIV-1/HCV co-infected patients, and blue and green indicated nucleus and Nef protein, respectively.

Next, we investigated whether Nef protein can be transferred from HIV-1-infected cells to hepatocytes. To this end, when over 95% Jurkat cells supported HIV-1 replication based on p24 analysis, the infected cells were mixed with RLuc cells, and transfer of Nef protein from the infected Jurkat cells to RLuc was examined by confocal microscopy using anti-nef antibody followed by anti-mouse Alexa488. Jurkat cells harboring Δnef-HIV-1 did not exhibit a green color (Fig. 3A, left), whereas wild type HIV-1-infected Jurkat cells did (Fig. 3A, right), reflecting the presence and absence of Nef protein, respectively. Analysis of the confocal images showed that an average of 16.4% RLuc was green+/red+, when RLuc was co-cultured with the wild type HIV-1-infected Jurkat cells, while only 3.6% RLuc was green+/red+, when the cells were co-cultured with dnef-HIV-1-infected Jurkat cells, indicating that Nef protein expressed in HIV-1 replicating cells can be transferred to the target cells (Fig. 3A, right). Next, we investigated whether the transfer of Nef from virus infected cells to primary hepatocytes takes place to confirm that the observed Nef transfer above is not an in vitro artifact. To this end, we obtained a paraffin-embedded hepatocyte specimen from HCV mono-infected as well as HIV-1/HCV co-infected patients, and the specimen was stained with anti-Nef antibody followed by HRP-conjugated secondary antibody and tyramide labeling to assess Nef transferal. IHC analysis showed that Nef protein was indeed detectable in liver samples obtained from co-infected (Fig. 3B, right) but not from HCV mono-infected patients (Fig. 3B, left), indicating that Nef protein was transferred to hepatocytes in co-infected patients.

### HIV-1 Nef transferred to the target subgenomic replicon cells was sufficient to augment HCV replication

**Figure 4:**
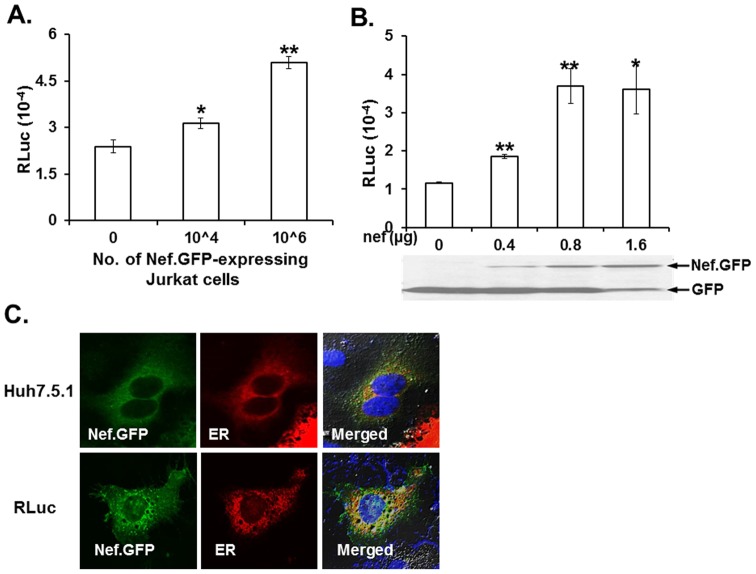
Effect of Nef on HCV replication. (A) Effect of co-cultivation of nef-expressing Jurkat with RLuc on the expression of replicon. The indicated number of nef.GFP-expressing Jurkat was co-cultured with RLuc, and the effect of Nef on replicon expression was assayed by measuring RLuc activity. “0” in the X-axis represents the control of GFP-expressing Jurkat cells (10∧6), and the total number of Jurkat was adjusted with GFP-expressing Jurkat to be 10∧6 cells. The data were expressed as mean +/− SEM of triplicates. (B) (upper panel) Effect of Nef on the expression of HCV subgenomic replicon. Replicon cells were transfected with the indicated amount of nef.GFP-expressing plasmid. “0” in the X-axis indicates the amount of GFP-expressing plasmid (1.6 µg), and GFP-expressing plasmid input was adjusted to equalize total plasmid transfected. Forty-eight hours post-transfection, cells were harvested and Renillar luciferase (RLuc) activity was assayed. Means and standard deviation values of triplicate experiments are depicted as bars and lines. (Lower panel) Western blot analysis. RLuc replicon cells were cultured onto a 6 well plate and transfected with the indicated amount of nef.GFP-expressing plasmid under similar conditions as above. Cell lysates were prepared, and expression of GFP or Nef.GFP fusion protein was detected by Western blot analysis using anti-rabbit anti-GFP antibody. GFP and Nef.GFP are indicated with arrows. (C) Subcellular localization of Nef. Huh7.5.1 or RLuc cells were co-transfected with plasmids to examine the expression of nef.GFP fusion protein and an ER marker, and subcellular localization of Nef was analyzed by immunofluorescence microscopy. A portion of Nef protein (green) was localized to the ER (red), depicted in yellow when the two colors were merged.

The next question that we addressed was whether Nef protein transferred from expressing cells is sufficient to up-regulate HCV replication to expedite HCV-mediated liver disease progression. To answer this question, we investigated the impact of co-cultivation of nef-expressing Jurkat cells with RLuc on the replicon expression. Our data clearly showed that replicon expression was enhanced, given increased numbers of nef.GFP-, but not GFP-expressing Jurkat cells, indicating that the transferred Nef was sufficient to enhance HCV replication (Fig. 4A). In corroboration with the above data (Fig. 2), no subgenomic replicon expression was detected when RLuc was cultured with the exosomal fraction or culture supernatants generated from nef.GFP-expressing Jurkat cells (data not shown), as shown in Fig. 2, confirming that transfer of Nef via exosomes was negligible. Taken together, these data indicated that Nef transferred to the target subgenomic replicon cells could be sufficient to augment HCV replication so as to facilitate disease progression in the co-infected patients. Consistent with these data, transfection of the HIV-1 nef.GFP gene increased replicon expression by 2∼4 fold in a dose dependent manner, which was examined by measuring reporter gene (Luc) expression in replicon cells (Fig. 4B). In contrast, transfection of the tat-expressing plasmid did not show any enhancement of HCV replication even at high concentrations (data not shown), demonstrating the specificity and significance of Nef, not Tat, in the enhancement of HCV replication by co-infection. It was important to next resolve how Nef, which has been localized to the cytoplasmic membrane [12], [47], can up-regulate HCV replication which takes place in the lipid-rich subcellular milieu of the ER [48], [49], [50], [51], [52]. To address this issue, we examined the subcellular localization of Nef in hepatocytes by co-transfecting plasmids for expression of the nef.GFP fusion protein, along with an ER marker. Our data demonstrated that a portion of the Nef protein (green) was indeed localized to the ER (red), indicated in yellow, which resulted when both colors were merged in Huh7.5.1 cells (Fig. 4C). Similar results were obtained using RLuc replicon cells (lower panel, Fig. 4C). Taken together, these data indicate that Nef can up-regulate HCV replication in the ER.

### Nef altered LD formation

**Figure 5:**
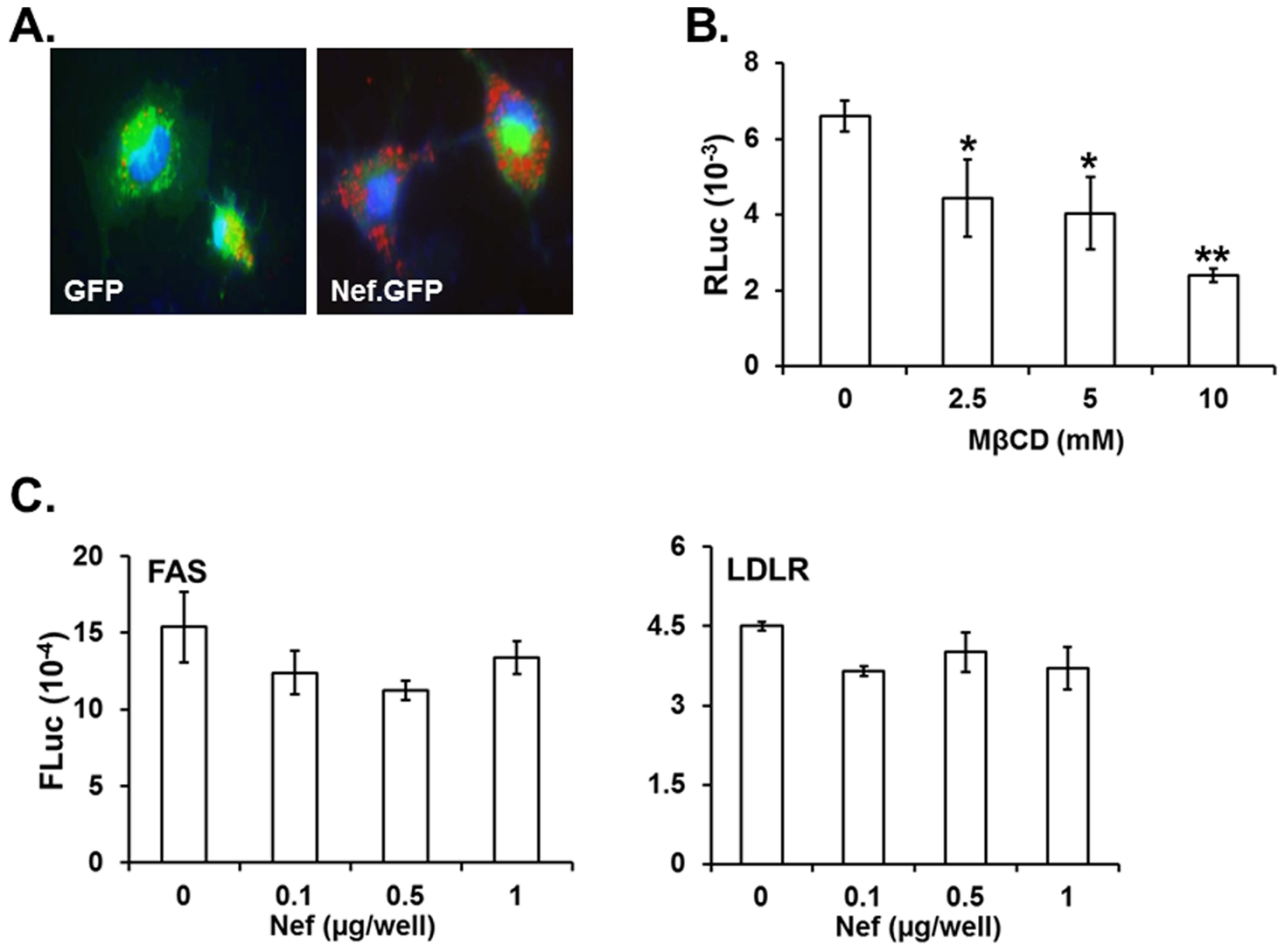
Effect of Nef on the expression of lipids and the formation of LD. (A) Formation of LD. RLuc cells cultured on a cover slip were transfected with gfp- or nef.GFP-expressing plasmids, and the transfected cells were stained with ORO (red). (B) Effect of MβCD on Nef-mediated alteration of replicon expression. RLuc cells transfected with nef.GFP-expressing plasmid were treated with the SF-DMEM for 24 hr, followed by a 1 hr treatment with the indicated concentration of MβCD in the SF-DMEM. RLuc activity was then determined in the cells. Means and standard deviation values of triplicate experiments are depicted as bars and lines. (C) Effect of Nef on the promoter activities for the lipid synthesis genes. Each promoter-reporter construct, pGL2B-FAS-1500 (FAS) or LDLRLuc (LDLR), was co-transfected with the indicated amount of gfp- or nef.GFP-expressing plasmids, and at 48 hr post-transfection, a FLuc assay was performed with the cell lysates from the transfected cells using a *Firefly* Luciferase assay kit.

To identify Nef-mediated changes in HCV replication and their consequent regulation of hepatocellular biology, we expressed the nef gene in hepatocytic cell lines by transfection, instead of using a co-culturing system to allow Nef transfer, since transfection efficiency is much higher than nef transfer (10%), as shown in Fig. 4, and therefore physiological changes in the system will be more pronounced to ameliorate detection of the Nef-mediated changes. It is reported that HIV-1 Nef modulates the distribution and levels of lipids [53], [54], nef-mediated enhancement of HCV replication might result from changes in the size and number of LD at the HCV replication site. Thus, this possibility was investigated by staining LD with ORO in RLuc cells transfected with gfp- or nef.GFP-expressing plasmid. The results indicated a significant increase in number and size of LD in nef.GFP-expressing RLuc cells, compared with gfp-expressing RLuc cells (Fig. 5A), suggesting that alterations of the LD in the presence of HIV-1 nef provides a better cellular environment for the up-regulation of HCV replication. These results indicate that nef-mediated-up-regulation of HCV replication could be the result of the modulation of lipid molecules at the replication site of HCV. To elucidate further whether the modulation of LD contributed to the nef-mediated enhancement of HCV replication, nef-expressing RLuc cells were treated with MβCD, which sequesters lipids at the cyclodextrin ring without changing level of the expression of intracellular lipid molecules [55], and the effect of the agent on the nef.GFP-mediated alteration of HCV replication was determined in the replicon system. Our data showed that replication of HCV by Nef was decremented, as the concentration of MβCD was increased (Fig. 5B). These results indicate that nef-mediated-up-regulation of HCV replication could be the result of the modulation of lipid molecules at the replication site of HCV. However, these data did not reveal whether the observed increases in size and number of LD were due to the increases of total amount of lipid molecules in the cells or to the redistribution of intracellular lipid molecules, so that the optimal subcellular environment is provided for efficient HCV replication. To answer this question, the nef.GFP-expressing plasmid was co-transfected with the indicated amount of the promoter-luciferase reporter plasmid for fatty acid synthase (FAS) and for low-density lipoprotein receptor (LDLR), and the effect of Nef on these promoter activities was investigated. The data showed that Nef did not exert any distinct changes in these promoter activities (Fig. 5C). Consistent with this, nef expression did not change the amount of intracellular cholesterol (data not shown). Furthermore, up-regulation of the sterol regulatory element binding protein-1 (SREBP-1), a family of transcription factor that regulates lipid homeostasis by controlling the expression of a range of enzymes required for endogenous cholesterol, fatty acid, triacylglycerol and phospholipid synthesis [56], [57], [58], was not detected even at the highest concentration of Nef (data not shown), confirming that Nef did not up-regulate the amount of the tested intracellular lipid molecules. Taken together, these data suggest that alterations in LD were not a consequence of Nef-mediated up-regulation, but an outcome of redistribution of intracellular lipid molecules.

### Ethanol treatment of nef-expressing RLuc cells showed an additive effect on HCV replication

**Figure 6:**
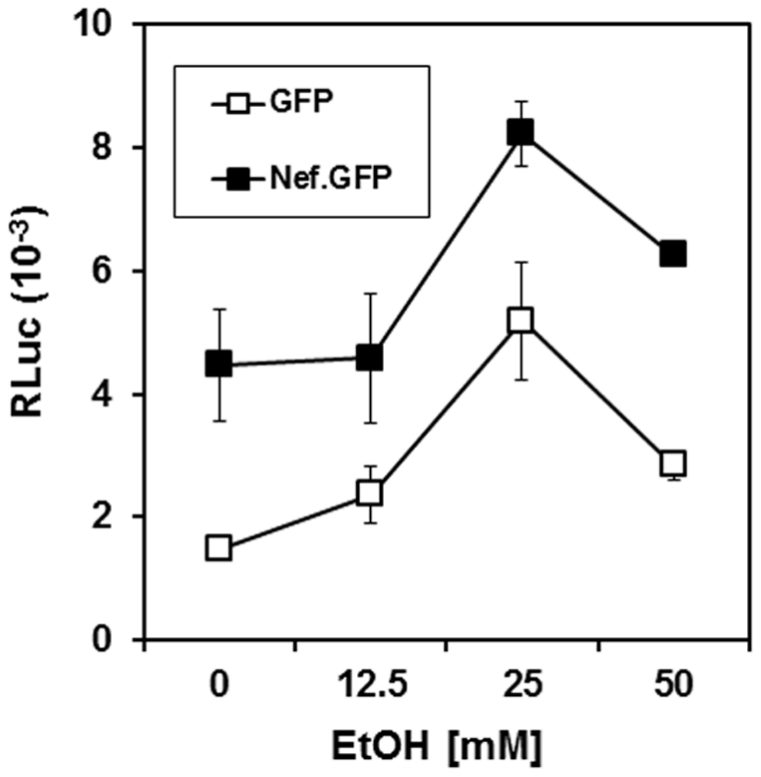
Effect of ethanol on nef-mediated alteration of HCV replicon expression. RLuc cells were transfected with gfp- or nef.GFP-expressing plasmids, and the cells were treated with the indicated concentration of ethanol for 48 hr. Renillar luciferase activity in each transfected lysate was then measured. Means and standard deviation values of triplicate experiments are depicted as lines.

It is very well known that administration of ethanol enhances HCV replication [59]. Thus, we investigated whether the nef-mediated up-regulation mechanism of HCV replication is related to alcohol-induced alterations in HCV replication. To this end, RLuc cells were transfected with nef-expressing plasmid, and the transfected cells were treated with the indicated concentration of ethanol to analyze changes in replicon expression. Our data showed that consistent with the previous report [59], treatment of absolute ethanol enhanced HCV replication, peaking at ethanol concentrations of 25 mM. When the nef-expressing cells were treated with ethanol, HCV replication was additively augmented, (Fig. 6). The additive effect of both ethanol and HIV-1 Nef on HCV replication suggests that both may partially share similar signaling cascades leading to increases in HCV replication.

### Nef augmented ROS production

**Figure 7:**
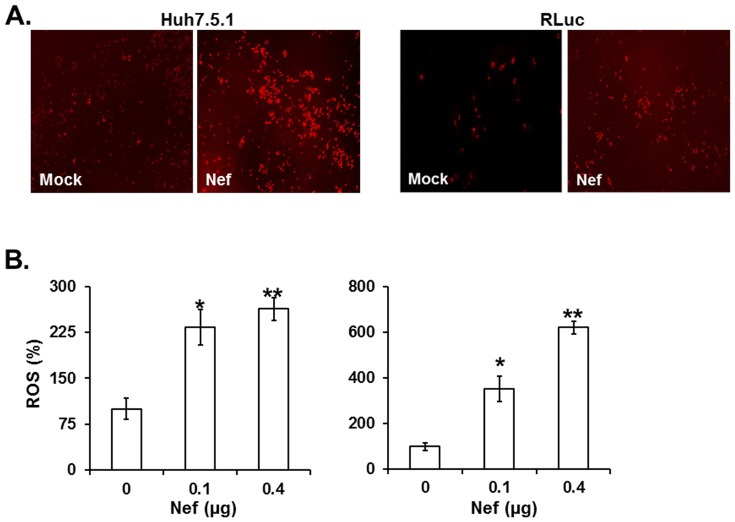
Analysis of ROS. (A) MitoSox Red staining. Huh7.5.1 (left) or RLuc (right) cells transfected with 0.1 µg of isotype (mock) and nef-expressing plasmid (Nef) were stained with MitoSox Red and visualized by fluorescence microscopy (x5). (B) Quantification of the ROS. Huh7.5.1 (left) and RLuc (right) were transfected with the indicated amount of nef-expressing plasmid in sextuplicate and loaded with DCFDA after 48 h culture. Fluorescence was measured at 495 nm excitation and 530 nm emission wavelengths, and the relative amount of ROS was shown.

ROS is a critical regulator of hepatic fibrosis progression [60], [61], [62], [63], [64], whose level is increased by ethanol metabolism and HCV infection [60], [65], [66], [67]. It is also reported that HIV-1 and HCV cooperatively promotes hepatic fibrogenesis by inducing ROS [13], [68], and therefore we investigated whether Nef is a responsible element in generation of ROS in the hepatocytes. MitoSox Red staining data indicated that Nef significantly augmented generation of superoxide in both Huh7.5.1 (left panel) and RLuc (right panel, Fig. 7A). Consistent with the previous data that HCV infection induces ROS generation (34, 48), our data showed that Nef dramatically enhanced ROS generation measured by DCFDA intensity (Fig. 7B) and the increase of ROS generation was much higher in subgenomic replicon, RLuc cells than in Huh7.5.1 (6 fold vs 3 fold) (Fig. 7B), confirming that HCV viral proteins contributed to production of ROS.

## Discussion

Our data demonstrate that HIV-1 Nef is transferred from HIV-1 infected cells to HCV subgenomic replicon cells, wherein Nef protein enhances HCV replication, most likely by making changes in LD formation and distribution. Further, treatment of a nef-expressing subgenomic replicon with absolute ethanol resulted in an additive effect on HCV replication. Nef also dramatically increased ROS generation, whose level is also enhanced by ethanol metabolism and HCV infection (16, 34, 48, 54), indicating that Nef could play an essential role in HIV-1-mediated acceleration of liver disease progression in different molecular mechanisms.

Previous reports indicated that HIV-1 was capable of infecting hepatocytes [14], [15], [16], [18] and suggested that replication of HIV-1 in liver cells may contribute to an acceleration of the progression of liver diseases caused by HCV infection. In contrast to these reports, other study indicates that HIV-1 can modulate HCV replication in hepatocytes in the absence of productive HIV-1 replication by inducing the secretion of TGF-β1 [13]. Similarly, our data demonstrated that HIV-1 neither enters into nor replicates within the hepatic cell line or in the HCV replicon cells, as shown in Fig. 1. More importantly, co-cultivation of Jurkat T cells with the transfected hepatic cell lines did not show any sign of HIV-1 replication, demonstrating that acceleration of liver disease by co-infection might not be due to HIV-1 replication itself in the hepatocytes.

Thus, advances of liver disease by HIV-1 co-infection could result from interactions between HIV-1 viral proteins and cellular and/or viral proteins of HCV infected cells, rather than replication of HIV-1 in hepatocytes. It is indeed reported that HIV-1 viral proteins are involved in regulation of HCV replication, which in turn changes the pathologic course in HCV infected patients. Specifically, Env of HIV-1 triggers signaling cascades by interacting with surface molecules, such as CXCR4 of hepatocytes, thus enhancing HCV replication [25], inducing apoptosis of hepatocytes, or causing hepatic inflammation [26], [27], [28], [29]. HIV-1 viral protein Tat, could be diffused from HIV-1 infected cells to hepatocytes and cause hepatocellular carcinoma (HCC), which is supported by a report that Tat itself enhances hepatocarcinogenesis in transgenic mice [21], [22]. Interestingly, a recent report indicates that Vpu protein degrades NS4A to release NS3 from the NS3/4A complex for nuclear translocation, wherein NS3 stimulates HIV-1 LTR transcription [69].

Unlike these viral proteins, HIV-1 Nef induces the protrusion of conduits [30], [31] and the transfer of expressed Nef protein to target cells via these conduits and, as a result, disturbs normal functions of target cell biology (47, 69). Consistent with these reports, our data demonstrated that HIV-1 Nef was transferred from Jurkat cells to RLuc cells through conduits, not through exosomes, and up-regulated HCV replication therein. Nef transfer was further confirmed by co-cultivation of HIV-1-infected Jurkat cells with RLuc (Fig. 3). The transferred Nef was detected in RLuc, when the cells were co-cultivated with wt- but not with Δnef-HIV-1-infected Jurkat cells, indicating that Nef from infected cells can also be transferred to hepatocytic cells (Fig. 3). Furthermore, Nef protein was detected in liver samples obtained from co-infected (Fig. 3) but not from HCV-mono infected patients (Fig. 3), indicating that the observed transfer of Nef to hepatocytes above is not an in vitro artifact.

Our data indicate that the transferred Nef could expedite liver disease progression by up-regulating HCV replication. Specifically, the transferred Nef protein in RLuc enhanced the number and size of LD, as shown in Fig. 5, and an increase in HCV replication by Nef was obliterated when the cells were treated with MβCD, which sequesters lipid molecules into its cyclodextrin ring (Fig. 5), suggesting that nef-mediated augmentation of HCV replication could be due to changes in lipid distribution at the HCV replication sites. This interpretation is reasonable, in light of previous reports that HCV requires lipid molecules for an efficient replication [70], [71] and that simian immunodeficiency virus (SIV) Nef protein was detected in the liver of the SIV-infected macaques, whereas the Nef protein down-regulated the cholesterol transporter ABCA1 [72]. In addition to sequestering lipid molecules into the cyclodextrin ring, MβCD could impede Nef transfer from expressing cells to the target hepatic cell lines and thereby nullify Nef-mediated enhancement of HCV replication, inasmuch as the compound is known to be targeted to the lipid rafts [73], [74] so as to hinder the presence of Nef in the lipid rafts at the plasma membrane. Hence, whether obliteration of Nef-mediated augmentation of HCV replication by MβCD was due to deprivation of lipid molecules in the ER where HCV replication takes place and/or impairment of Nef transfer to the target hepatic cell lines remains to be resolved. Further, recent investigation has shown that HIV-1 Nef can directly impact the ER [75], whose effect on Nef-mediated modulation of HCV replication also needs to be elucidated.

Our data also show that Nef together with ethanol increases HCV replication in an additive manner (Fig. 6), suggesting that signaling cascades triggered by ethanol or by Nef could be shared at least partially. According to the previous study, alcohol augmented HCV replicon expression by activating nuclear factor κB (NFκB) [59] which is induced by protein kinase Cθ, a crucial signaling element for HCV replication [76]. It is also known that HIV-1 Nef activates Lck which targets protein kinase Cθ [77], leading to the translocation of the kinase into membrane microdomains [78] and that Nef modulates lipid composition in the membrane microdomains by triggering various signaling molecules [53], [79], [80]. Thus, studies on the cooperative regulation mechanisms of signaling cascades by Nef and by alcohol will greatly enhance our understanding of pathobiology of liver diseases.

Finally, Nef is known to regulate generation of superoxide anions from human macrophages [81], [82], [83] and increase astrocyte sensitivity towards exogenous hygrogen peroxide [83], [84], [85]. However, it has not been known that Nef is a responsible viral element in regulation of ROS production in hepatocytes. Our data indicated that Nef augmented generation of superoxide and dramatically induced production of ROS (Fig. 7). Consistent with the previous data that HCV infection induces ROS production [60], [65], [66], [67], our data showed that ROS production was much higher in subgenomic replicon RLuc cells than in Huh7.5.1 (6 fold vs 3 fold) (Fig. 7), confirming that HCV viral proteins contribute to production of ROS. Since Nef enhanced generation of ROS, up-regulated HCV replication, and additively promoted ethanol-mediated HCV replication, these data indicate that Nef-mediated aggravation of liver diseases by ROS production can be multiplied by ethanol and/or by augmentation of HCV replication. Further, in light of the previous report that ROS induces production of cytokine, such as TGFβ1, for promotion of hepatic fibrogenesis [64], Nef could facilitate liver disease progression by enhancing secretion of the cytokine via ROS production.

**Figure 8:**
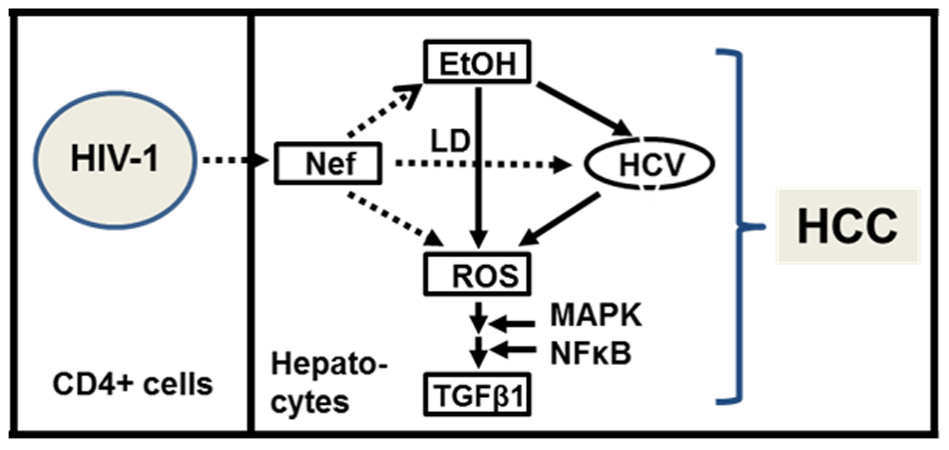
Schematic presentation of Nef role. The transferred Nef from HIV-1-infected cells to hepatocytes through conduits could exacerbate liver decay by enhancing HCV replication independently or together with ethanol and by producing ROS independently or synergistically with ethanol/HCV replication cycle. The dashed arrows indicate newly identified Nef role in hepatocytes.

Taken together, these data indicate that the transferred Nef from HIV-1-infected cells to hepatocytes through conduits is capable of exacerbating liver decay by enhancing HCV replication and by synergizing the ROS/HCV and/or ethanol/HCV replication cycle, where alcohol is known to be the second most important causative agent for HCC [86], as depicted in Fig. 8. However, molecular mechanisms for how Nef leads to these changes are unclear. It is reported that Nef interacts with HCV core [43], which could exert changes on the observed biological actions, since both Nef and HCV core protein activate tumor necrosis factor receptor-associated factor which plays a central role in regulation of many biological activities, such as immune and inflammatory responses and apotosis [43]. Further, since HCV core protein is also known to be linked to the formation of LD clusters [87], it is reasonable to suggest that Nef-mediated alteration of LD formation together with HCV core is critical for providing an optimal subcellular milieu for an efficient HCV replication. Nef could also trigger signaling cascades to activate key intracellular molecules, as discussed above, and this possibility deserves investigation. Elucidation of mechanisms behind the observed data will enhance our understanding of the pathobiology of co-infection and provide important clues to develop therapeutics against liver diseases in co-infected patients.
